# Real-world evidence of cytomegalovirus reactivation in non-Hodgkin lymphomas treated with bendamustine-containing regimens

**DOI:** 10.1515/med-2021-0274

**Published:** 2021-04-21

**Authors:** Luca Pezzullo, Valentina Giudice, Bianca Serio, Raffaele Fontana, Roberto Guariglia, Maria Carmen Martorelli, Idalucia Ferrara, Laura Mettivier, Alessandro Bruno, Rosario Bianco, Emilia Vaccaro, Pasquale Pagliano, Nunzia Montuori, Amelia Filippelli, Carmine Selleri

**Affiliations:** Hematology and Transplant Center, University Hospital “San Giovanni di Dio e Ruggi d’Aragona”, Salerno, 84131, Italy; Clinical Pharmacology, University Hospital “San Giovanni di Dio e Ruggi d’Aragona”, Salerno, 84131, Italy; Department of Medicine, Surgery and Dentistry “Scuola Medica Salernitana”, University of Salerno, Baronissi, 84081, Salerno, Italy; Transfusion Medicine, Molecular Biology Section, University Hospital “San Giovanni di Dio e Ruggi d’Aragona”, Salerno, 84131, Italy; Infectious Disease Unit, University Hospital “San Giovanni di Dio e Ruggi d’Aragona”, Salerno, 84131, Italy; Department of Translational Medical Sciences, “Federico II” University, 80138, Naples, Italy

**Keywords:** cytomegalovirus, non-Hodgkin lymphoma, chemotherapy, immunity

## Abstract

Cytomegalovirus (CMV) reactivation during chemotherapy or after organ or hematopoietic stem cell transplantation is a major cause of morbidity and mortality, and the risk of reactivation increases with patients’ age. Bendamustine, an alkylating agent currently used for treatment of indolent and aggressive non-Hodgkin lymphomas, can augment the risk of secondary infections including CMV reactivation. In this real-world study, we described an increased incidence of CMV reactivation in older adults (age >60 years old) with newly diagnosed and relapsed/refractory indolent and aggressive diseases treated with bendamustine-containing regimens. In particular, patients who received bendamustine plus rituximab and dexamethasone were at higher risk of CMV reactivation, especially when administered as first-line therapy and after the third course of bendamustine. In addition, patients with CMV reactivation showed a significant depression of circulating CD4^+^ T cell count and anti-CMV IgG levels during active infection, suggesting an impairment of immune system functions which are not able to properly face viral reactivation. Therefore, a close and early monitoring of clinical and laboratory findings might improve clinical management and outcome of non-Hodgkin lymphoma patients by preventing the development of CMV disease in a subgroup of subjects treated with bendamustine more susceptible to viral reactivation.

## 1 Introduction

Non-Hodgkin lymphomas (NHL) are a heterogeneous group of hematologic diseases with various biological and clinical features classified in B, NK, or T cell lymphomas with different morphology, immunophenotype, genetic, and clinical features [1,2,3]. Diffuse large B-cell lymphoma (DLBCL) and Chronic Lymphocytic Leukemia (CLL) are the most common B-cell NHL, while mantle cell lymphoma (MCL) is less frequent (3% of cases) [1,4]. Prognosis varies for each entity also based on clinical and molecular signatures. For example, in DLBCL, a mature B cell NHL frequently affecting older people, five-year overall survival (OS) ranges from 36 to 67% depending on nodal or extranodal involvement and combination of standard chemotherapy with rituximab [4,5,6]. In CLL, five-year OS can vary from 23.3 to 93.2% based on disease stage and serum markers [7,8,9]. Similarly, in follicular lymphoma (FL), the second most diagnosed lymphoma in Western Countries and frequently occurring in older patients, prognosis is influenced by clinical features, such as disease stage or number of involved nodal areas and two-year OS varies from 87% in high-risk to 98% in low-risk patients [10].

Bendamustine, an alkylating chemotherapeutic agent consisting of a purine analog-like benzimidazole ring, an alkylating agent group, and an alkane carboxylic chain, causes more durable intra- and inter-strand DNA cross-links than other alkylators and induces DNA damage and mitotic catastrophe [11,12,13]. Bendamustine is currently approved as first-line treatment of CLL Binet stage B or C, indolent NHL (iNHL) as monotherapy in patients relapsed or refractory to rituximab-based chemotherapy, and as first-line treatment in multiple myeloma patients not eligible for autologous stem cell transplantation [11]. This chemotherapeutic agent has also been used in newly diagnosed or relapsed/refractory DLBCL and T-cell NHL [14,15,16,17]. Bendamustine-containing regimens are well-tolerated. The most frequent toxicities are thrombocytopenia, lymphopenia, an increased risk of infections by opportunistic pathogens, such as *Pneumocystis jirovecii*, and higher risk of reactivation of chronic viral infections, including cytomegalovirus (CMV), Epstein-Barr, and Varicella-Zoster virus (VZV) [18,19,20]. CMV, a human herpesvirus, has an estimated seroprevalence worldwide of 45–100% in general population, and primary infection always occurs asymptomatically in immunocompetent subjects; however, immunocompromised patients experience a more aggressive disease with fever, hepatitis, severe pneumonia, encephalopathy and polyradiculopathy, myelosuppression, and graft rejection [21,22].

Bendamustine alone and in association with other immunosuppressive agents, such as steroids and rituximab, might increase the risk of CMV reactivation; however, literature lacks prospective longitudinal studies in large cohorts. In this real-world study, we investigated incidence of CMV reactivation in 167 consecutive patients with indolent and aggressive lymphoproliferative disorders treated with bendamustine-containing regimens, and candidate biomarkers of viral reactivation were also explored.

## 2 Subjects and methods

### 2.1 Patients and therapeutic regimens

Patients received diagnosis and chemotherapy as per international guidelines after informed consent obtained in accordance with the Declaration of Helsinki at the Hematology and Transplant Center, University Hospital “San Giovanni di Dio e Ruggi d’Aragona” of Salerno, Italy, from June 2010 to April 2020. For assessment of CMV reactivation, a total of 167 patients with diagnosis of NHL were included in this retrospective study. Inclusion criteria were: age >18 years old; diagnosis of NHL; and treatment with bendamustine alone or in combination as first or above line of therapy. Median age was 70 years old (range, 41–88) and males were 59% (*N* = 99) (Table 1). NHL was diagnosed following the 2008 or 2016 revision of the World Health Organization classification of lymphoid neoplasms [1,23]; in particular, 95% (*N* = 158) B-cell NHL: DLBCL (*N* = 38; 23%); FL (*N* = 39; 23%); CLL/small lymphocytic lymphoma (SLL; *N* = 32; 19%); mantle cell lymphoma (MCL; *N* = 17; 10%); marginal zone lymphoma (MZL; *N* = 18; 11%); lymphoplasmacytic lymphoma (PLP; *N* = 4); plasmablastic lymphoma (PbL; *N* = 1); B-cell NHL not otherwise specified (NOS; *N* = 4); mucosa-associated lymphoid tissue (MALT) lymphoma (*N* = 4); and acute lymphoblastic leukemia (ALL; *N* = 1). Two subjects were diagnosed with Hodgkin’s lymphoma, one with multiple myeloma, one with Waldenstrom disease, and 3% (*N* = 5) of patients with T-cell NHL.

**Table 1:** Baseline patients’ characteristics

Characteristics	*N* = 167
Age, years	70 (41–88)
Sex, M/F	99/68
**Diagnosis**	
DLBCL	38
FL	39
CLL/SLL	32
MCL	17
MZL	18
Other B-cell NHL	14
T-cell NHL	5
Others	4
**Stage**	
I	4
II	21
III	23
IV	82
**First-line therapy**	
Bendamustine-based	138
Standard chemotherapy	20
Stem cell transplantation	2
No treatment	7
**Second-line therapy**	
Bendamustine-based	18
Standard chemotherapy	3
>2 lines of chemotherapy	12
Median cycles of bendamustine	6 (1–10)
Median follow-up, months	15.3 (0.6–99.5)
**CMV serology**	
IgG^−^	4
IgG^+^	80

Abbreviations: DLBCL, diffuse large B cell lymphoma; FL, follicular lymphoma; CLL, chronic lymphocyte leukemia; SLL, small lymphocytic lymphoma; MCL, mantle cell lymphoma; MZL, marginal zone lymphoma; NHL, non-Hodgkin lymphoma; CMV, cytomegalovirus; IgG, immunoglobulin G.

Patients received bendamustine in combination with rituximab (RB regimen), rituximab and/or dexamethasone (DB, dexamethasone plus bendamustine; RDB, rituximab, dexamethasone, and bendamustine), and/or lenalidomide (LB, lenalidomide plus bendamustine; RLB, rituximab, lenalidomide, and bendamustine), or gemcitabine (GB). Bendamustine was given at doses of 70–90 mg/m^2^ for two consecutive days every 21 days for a maximum of four (RDB) or six (RB) cycles; while rituximab was administered at 375 mg/m^2^ every 21 days for a maximum of eight cycles, and dexamethasone was started at 20 mg/daily on days 1–4 and then tapered. Lenalidomide was administered at 25 mg/every other day. Patients with iNHL who achieved a complete remission received a maintenance therapy with rituximab at 375 mg/m^2^ every two months for two years. Therapeutic strategies non-containing bendamustine are summarized in Table 1. All patients received acyclovir and trimethoprim plus sulfamethoxazole as prophylaxis for VZV reactivation and for *Pneumocystis jirovecii* pneumonia, respectively.

### 2.2 Flow cytometry

**Figure 1:**
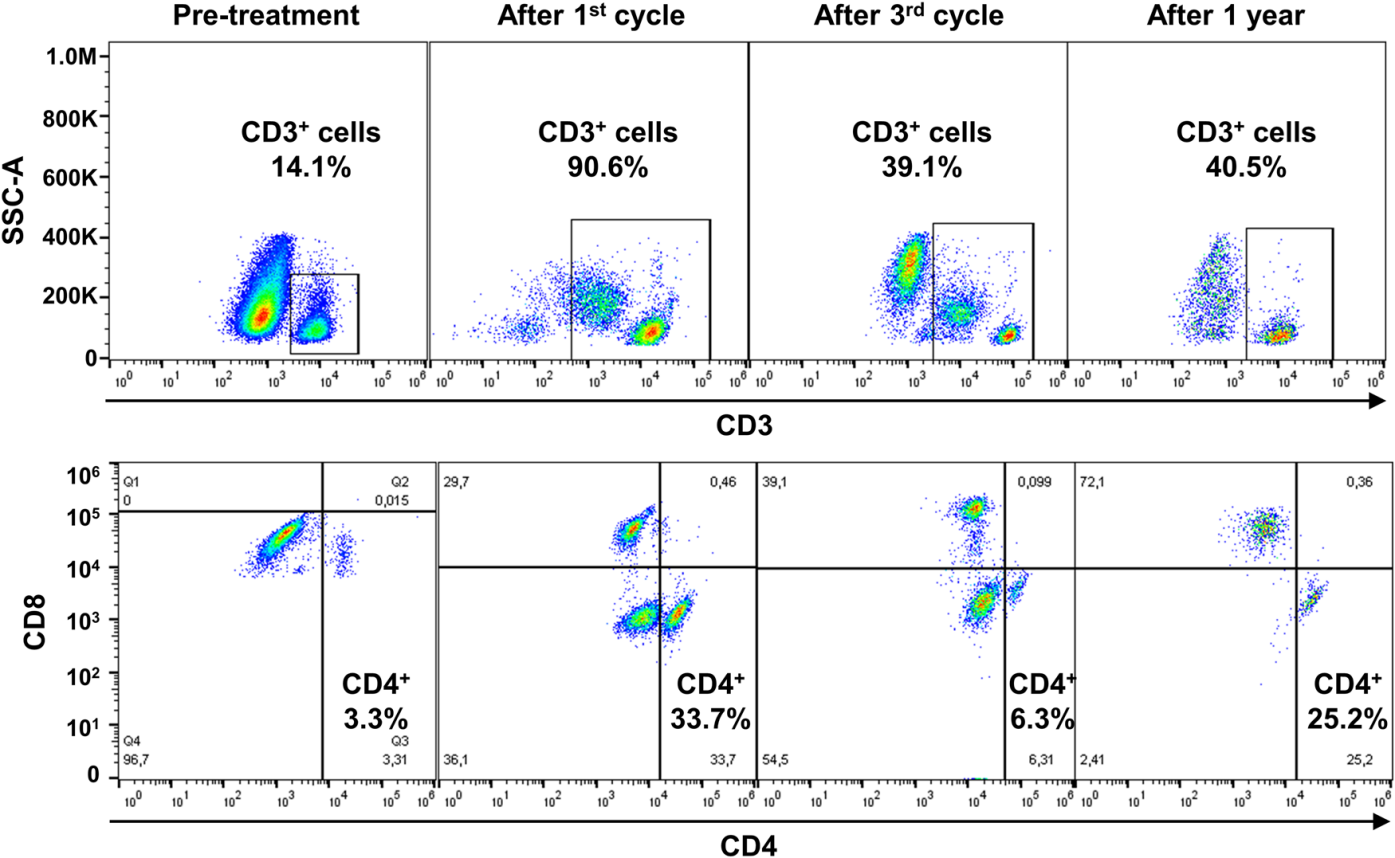
Flow cytometry gating strategy. After post-acquisition compensation using FlowJo, cell populations were first identified using linear parameters (forward scatter area [FSC-A] vs side scatter area [SSC-A], and double cells were excluded (FSC-A vs FSC-W)). On single cells, CD3^+^ cells were identified (CD3 vs SSC-A), and CD4 and CD8 expression was further studied. Flow cytometry analysis of a representative patient who experienced CMV reactivation is reported before starting treatment, after the first cycle of RDB and the third, and then after one year. Percent of CD3^+^ and CD4^+^ cells is shown for each timepoint.

Immunophenotyping was performed on fresh heparinized whole peripheral blood by flow cytometry (Figure 1). Neoplastic clones were identified using appropriate combinations of monoclonal antibodies as per manufacturer’s instructions (Beckman Coulter). CD4^+^ T cells were studied using the following antibodies: CD45; CD3; CD4; and CD8; and cell count assessed using beads as per manufacturer’s instructions (Beckman Coulter). Sample acquisition was carried out on a five-color FC500 cell analyzer cytometer (Beckman Coulter, Brea, CA, USA) or on a ten-color three-laser Beckman Coulter Navios Flow Cytometer (Beckman Coulter). At least 1 million events per sample were recorded. Post-acquisition analysis was performed using CPX or Navios tetra software (Beckman Coulter).

### 2.3 CMV-DNA quantification

Plasma CMV-DNA was quantified by real-time TaqMan CMV-DNA polymerase chain reaction (PCR) according to manufacturers’ instructions (Roche). During chemotherapy, CMV-DNA levels were measured every three weeks before starting each cycle, while CMV-DNA was monitored every week during CMV reactivation. After the end of treatment, CMV-DNA levels were measured every three months for two years. The instrument cut-off for positive results was CMV-DNA >137 copies/µL.

### 2.4 Statistical analysis

Data were collected in spreadsheet and analyzed using Prism (v.8.3.0; GraphPad software, San Diego, CA). Categorical variables were compared by Fisher exact test, while continuous variable using Mann-Whitney nonparametric test. Two-group comparison was carried out by unpaired *t*-test. Differences in cumulative incidence of CMV reactivation between groups were assessed by Log-rank (Mantel-Cox) test and Hazard Ratio (HR) by log-rank. Multivariate analysis was performed by logistic regression model using SPSS Statistics software (IBM, Armonk, NY). A *P* value of <0.05 was considered statistically significant.

## 3 Results

### 3.1 Patients characteristics at baseline

A total of 167 NHL patients were included in the study for assessment of CMV reactivation during bendamustine-based chemotherapy. At diagnosis, disease stage was evaluable in 130 out of 167 subjects: 3% of cases (*N* = 4) showed a stage I disease; 16% (*N* = 21) stage II; 18% (*N* = 23) stage III; and 63% (*N* = 82) stage IV disease. In addition, 44 patients (26%) showed involvement of extra lymphatic organ or site, especially gastrointestinal tract (*N* = 14). A total of 160 patients received a first-line chemotherapy and 138 of them (86%) were treated with a bendamustine-based regimen: two subjects (1% of total treated patients) with bendamustine as single agent; one (1%) with DB; four (3%) with GB; 107 patients (67%) received RB and 22 (14%) RDB; while 2 subjects (1%) with LB or RLB. The remaining 22 subjects did not receive bendamustine containing regimens as first-line therapy, and 19 out of these 22 patients did not achieve a partial or complete remission requiring RB (*N* = 16) or other regimens as second-line therapy. Two subjects nonresponsive to RB as first-line therapy received GB or RB with bendamustine at 90 mg/m^2^. Eleven subjects had a third-line therapy (three bendamustine alone; six RB; and two RLB) and one a fourth-line treatment (R-CHOP to R-GemOx to R-CHOP to RB). Median complete cycles with bendamustine were six (range, 1–10). The presence of specific IgG against CMV at baseline was assessed in 90 subjects, and 89% of them (*N* = 80) were positive for CMV-IgG. Median follow-up was 15.3 months (range, 19 days to 99.5 months).

### 3.2 Clinical characteristics of patients with CMV reactivation

Forty-one patients (25%) experienced CMV reactivation with a median time to reactivation after starting bendamustine of 54 days (range, 11–309 days). Median age of this group of patients was 69 years old (range, 54–86), and 66% were males. Patients with CMV reactivation had a diagnosis of B-cell NHL in 95% of cases (*N* = 39) and T-cell NHL in 5% of subjects (*N* = 2; two Sézary syndrome). Among B-cell NHL, 6 subjects had DLBCL (15%), 9 (22%) FL, 8 (20%) CLL/SLL, 6 (15%) MCL, 5 (12%) MZL, and the remaining 5 cases (12%) were diagnosed as following: three cases with MALT and two with PLP. At diagnosis, disease stage was evaluable in 35 out of 41 subjects: 10% of cases (*N* = 4) showed a stage II disease; 7% (*N* = 3) stage III; and 68% (*N* = 28) stage IV disease. One CLL subject received bendamustine plus rituximab as second-line therapy after failure of fludarabine plus alemtuzumab; an FL patient was treated with rituximab plus higher dose of bendamustine after partial response to rituximab plus bendamustine at 70 mg/m^2^; the two subjects with Sézary syndrome received bendamustine alone as third-line therapy after failure of interferon-based regimens, while one MZL subject received RB after nonresponsiveness to chlorambucil first and R-FluCy later. The remaining 36 patients were treated with RD in 78% of cases (*N* = 28) or RDB in 22% of cases (*N* = 8) as first-line therapy with a median bendamustine cycle of 7 (range, 2–8). At baseline, median anti-CMV IgG levels were 108 UI/mL (range, 42–180 UI/mL), median lymphocyte count was 1,340 cells/µL (range, 530–14,895), and median CD4^+^ T cell count was 617 cells/µL (range, 180–1,278). Clinical manifestations of CMV reactivation were present in 15 patients (37%) and were: fever (20%; *N* = 8); diarrhea (5%; *N* = 2); anemia (5%; *N* = 2); chorioretinitis (2%; *N* = 1); and mucositis (2%; *N* = 1). The nadir lymphocyte count was 365 cells/µL (range, 0–1,590 cells/µL), while nadir anti-CMV IgG levels were 532 UI/mL (range, 129–1,320), nadir anti-CMV IgM levels were 42.5 UI/mL (range, 19–190), and nadir anti-CMV IgA were 82 UI/mL (range, 24–410). Median CMV-DNA levels were 2,120 copies/µL (range, 151–2,230,000). The maximum peak of 2,230,000 copies/µL was described after 69 days of treatment with RB with bendamustine at 90 mg/m^2^ in a patient with FL. Valganciclovir (VGCV) was administered at 900 or 1,800 mg/daily or twice per week based on clinical symptoms. One patient received first intravenous (IV) immunoglobulins (Ig) and then VGCV at 900 mg/daily. A total of four CMV-related deaths occurred in our cohort of patients. A 54 years old male patient with a diagnosis of MZL, stage IVb, died because of severe CMV disease. Reactivation occurred within 11 days after initiation of RB, and VGCV first at 900 mg/daily and then IVIg were administered; however, he died because of nonresponsiveness to antiviral therapy and severe CMV disease. OS was 31.7 months in patients with CMV reactivation, while subjects who did not experience CMV reactivation had an OS of 81.3 months; however, there were no statistically significant differences between groups (*P* = 0.2929).

### 3.3 Predictors of CMV reactivation

**Figure 2:**
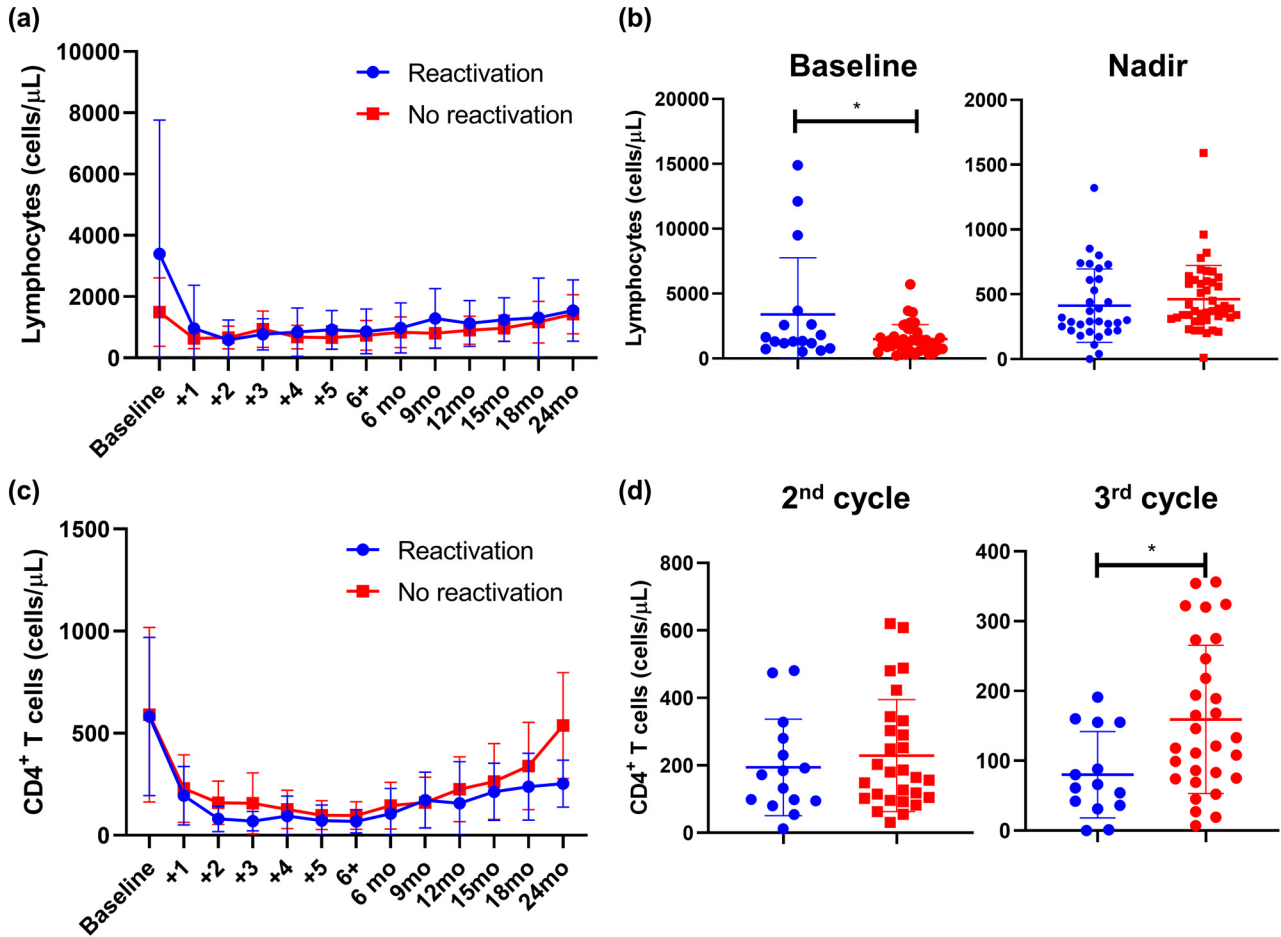
Lymphocyte and CD4^+^ T cell counts in CMV-reactivated and non-reactivated NHL patients. Patients were divided into two groups: subjects with CMV-reactivation (blue line or dots), and without CMV reactivation (red line or dots); and (a) lymphocyte counts are shown accordingly at baseline, before starting every cycle of bendamustine, and at regular monthly follow-up (month, mo). (b) Differences in lymphocyte counts at baseline and at the nadir of CMV reactivation were assessed by unpair *t*-test between patients who experienced CMV reactivation (blue dots) and those who did not have viral reactivation (blue dots). Similarly, CD4^+^ T cell counts were assessed from baseline through the follow-up (c), and frequencies of CD4^+^ cells in CMV-reactivated (blue dots) and non-reactivated (red dots) patients at the second and third cycle of bendamustine (d) are displayed. Data are shown as mean ± Standard Deviation (SD). *P < 0.05.

Whether to identify biomarkers of early CMV reactivation or risk factors, we compared clinical and laboratory findings between patients with CMV reactivation and subjects who did not experience viral reactivation. First, clinical and laboratory findings were compared between groups at baseline and during CMV reactivation (Table 2). No differences were described for age (*P* = 0.7069), number of bendamustine cycles (*P* = 0.6461), baseline anti-CMV IgG levels (mean ± SD, 111 ± 44.2 vs 113 ± 43.1, reactivation vs non-reactivation; *P* = 0.8610), and baseline CD4^+^ T cells (mean ± SD, 582 ± 387.8 vs 590 ± 427.5, reactivation vs non-reactivation; *P* = 0.9648). Significant variations were found in lymphocyte count at baseline (mean ± SD, 3,398 ± 4,366 vs 1,497 ± 1,117, reactivation vs no-reactivation; *P* = 0.0133), while frequencies were similar at the nadir of CMV reactivation (mean ± SD, 412 ± 282.9 vs 463 ± 259.7, reactivation vs non-reactivation; *P* = 0.4224) (Figure 2a and b). For CD4^+^ T cells, frequency similarly decreased after the first cycle of bendamustine in both CMV reactivated and non-reactivated groups; however, patients who did not experience CMV reactivation had higher CD4^+^ T cell count at the third bendamustine cycle compared to subjects who had CMV reactivation (mean ± SD, 80 ± 61.8 vs 159 ± 106.2, reactivation vs non-reactivation; *P* = 0.0133) (Figure 2c and d). In addition, anti-CMV IgG levels at the nadir of reactivation were lower in patients with CMV disease compared to patients who did not show viral reactivation (mean ± SD, 451 ± 208.7 vs 650 ± 254.6, reactivation vs non-reactivation; *P* = 0.0030), while anti-CMV IgA levels were similar between groups (*P* = 0.6259).

**Table 2:** Patients’ characteristics at CMV reactivation

Characteristics	CMV reactivation	No reactivation	*P* value
	*N* = 41	*N* = 126	
Age, years	69 (54–86)	71 (41–87)	0.7069
Sex, M/F	27/14	72/54	
Dead/Alive	17/24	46/80	
**Diagnosis**			
DLBCL	6	32	
FL	9	30	
CLL/SLL	8	24	
MCL	6	11	
MZL	5	13	
Other B-cell NHL	5	9	
T-cell NHL	2	3	
Others	—	4	
**Stage**			
I	0	4	
II	4	17	
III	3	20	
IV	28	54	
**First-line therapy**			
Bendamustine-based	37	101	
Standard chemotherapy	3	19	
**Second-line therapy**			
Bendamustine-based	2	16	
Standard chemotherapy	1	2	
>2 lines of chemotherapy	3	9	
Median cycles of bendamustine	7 (2–8)	6 (1–8)	0.6461
Median follow-up, months	11.5 (0.7–51)	18 (0.7–99.5)	***0.0097***
Time to reactivation, days	54 (11–309)	—	
Baseline CMV IgG, UI/mL	108 (42–180)	127 (25–180)	0.861
Baseline CD4^+^ T cells/µL	617 (180–1,278)	591 (118–1,761)	0.9648
Baseline lymphocytes/µL	1,340 (530–14,895)	1,180 (200–5,700)	***0.0133***
Nadir lymphocytes/µL	295 (0–1,320)	410 (230–820)	0.4224
Nadir CMV-IgG, UI/mL	412 (129–943)	670 (416–937)	***0.003***
CMV-IgM, UI/mL	38 (20–190)	—	
CMV-IgA, UI/mL	83 (24–410)	61 (49–158)	0.6259
CMV-DNA, copies/µL	2,030 (151–2,230,000)	—	
**Clinical manifestations**		—	
No symptoms	26		
Fever	8		
Diarrhea	2		
Anemia	2		
Chorioretinitis	1		
Mucositis	1		
Death	4		
**CMV treatment**			
Valgancyclovir	18		
IVIg	4		
Time to negativization, days	45.5 (17–152)		

Abbreviations: DLBCL, diffuse large B cell lymphoma; FL, follicular lymphoma; CLL, chronic lymphocyte leukemia; SLL, small lymphocytic lymphoma; MCL, mantle cell lymphoma; MZL, marginal zone lymphoma; NHL, non-Hodgkin lymphoma; CMV, cytomegalovirus; Ig, immunoglobulin; IVIg, intravenous immunoglobulins. *P* value < 0.05 was considered statistically significant and highlighted in bold italic.

**Figure 3:**
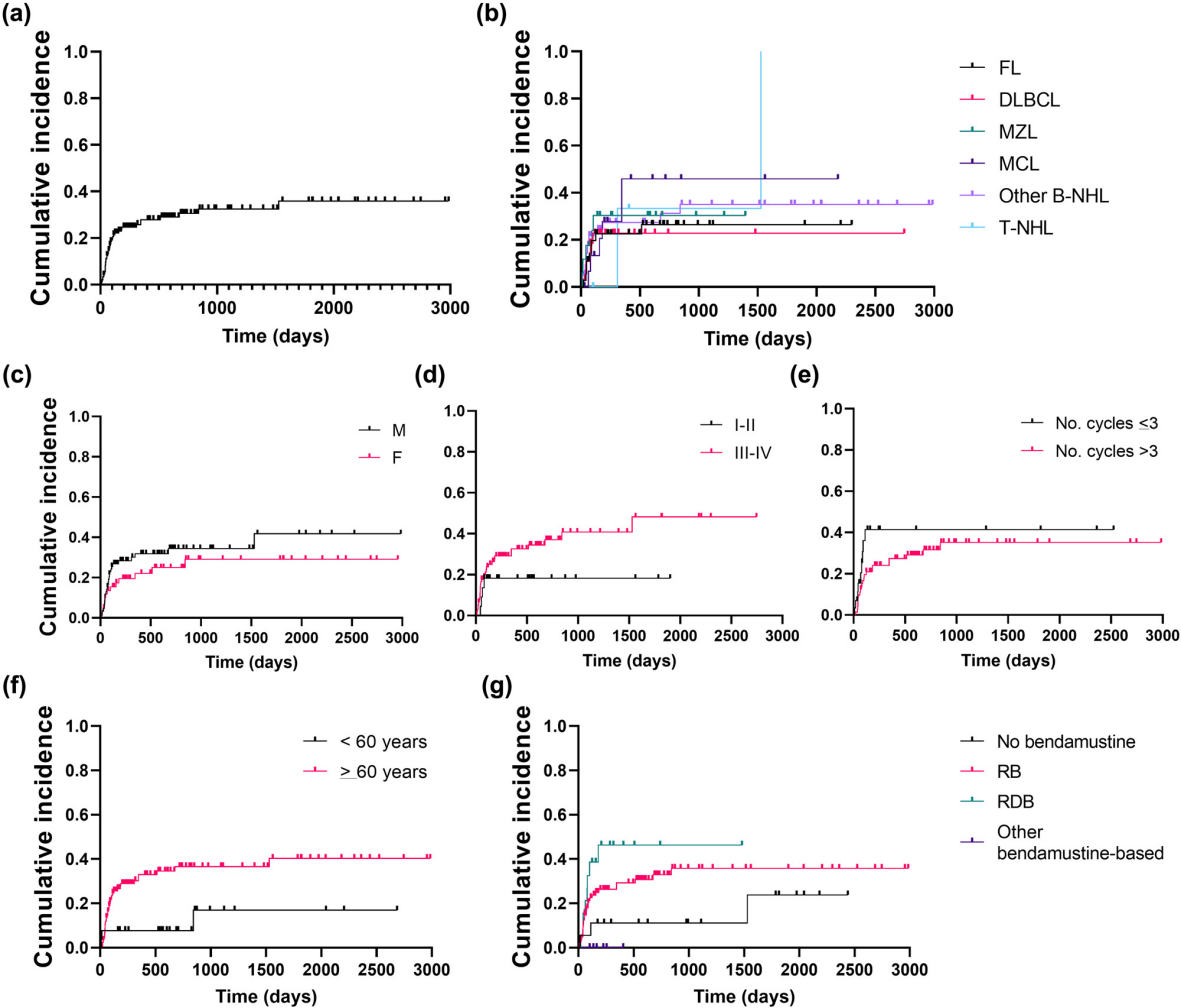
Cumulative incidence of CMV reactivation in NHL patients treated with bendamustine. (a) Cumulative incidence of CMV reactivation was first assessed on the entire cohort of patients diagnosed with NHL receiving bendamustine-containing regimens as first or above line of treatment. Then, influence of various clinical categories on the incidence of CMV reactivation was assessed by dividing patients based on: (b) type of lymphoma (FL, follicular lymphoma; DLBCL, diffuse large B cell lymphoma; MZL, marginal zone lymphoma; MCL, mantle cell lymphoma; other B-NHL, other B-cell non-Hodgkin lymphomas; T-NHL, T-cell non-Hodgkin lymphomas); (c) sex (M, male; F, female); (d) disease stage (stage I–II, and stage III–IV); (e) number (No.) of bendamustine cycles (≤3 or >3); (f) age (<60 years old [yo] or ≥60 years old); and (g) therapeutic regimens administered as first-line therapy (not-containing bendamustine; RB, rituximab plus bendamustine; RDB, rituximab plus dexamethasone and bendamustine; other bendamustine-containing regimens including the drug alone).

Next, cumulative incidence of CMV reactivation was calculated in our cohort of NHL patients showing an eight-year incidence of 46% (Figure 3a). Patients were then divided based on clinical and laboratory features and incidence proportion of CMV reactivation between groups was compared. No differences in CMV reactivation incidence were described when patients were divided based on sex (M vs F, *P* = 0.2607), diagnosis (*P* = 0.3963), disease stage (I–II vs III–IV, *P* = 0.1542), and number of bendamustine cycles (≤3 vs >3 cycles, *P* = 0.1753) (Figure 3b–e). Significant differences were described when patients were divided based on age with a cut-off of 60 years old (*P* = 0.0293; HR, 3.392; 95% confidential interval [CI], 1.602 to 7.180) and type of bendamustine-based regimen received as first-line therapy (Figure 3f and g). In particular, patients treated with RDB had a higher incidence of CMV reactivation compared to patients who did not receive bendamustine as first-line therapy (*P* = 0.0278; HR, 3.764; 95% CI, 1.134–12.49), while no differences were described between patients treated with RB or standard chemotherapy (*P* = 0.1658). Finally, multivariable analysis was performed in patients who received bendamustine-based regimens as first-line therapy and patients who were given standard chemotherapy as first-line treatment. In this latter group, none of analyzed variables, including age, sex, disease stage, cycles of bendamustine, nadir lymphocyte count, and anti-CMV IgG levels, was significantly associated to CMV reactivation, while in patients who received bendamustine as first-line treatment, potential risk factors were age ≥60 years old (*P* = 0.074), more than three bendamustine cycles (*P* = 0.027), and nadir anti-CMV IgG levels (*P* = 0.018).

## 4 Discussion

CMV reactivation in immunocompromised subjects can cause a life-threatening disease and NHL patients are at higher risk because immune responses are already impaired, and chemotherapy can worse this condition because of myelo- and immuno-suppression [22,24]. In this real-world study, incidence of CMV reactivation was systematically and retrospectively investigated in a large cohort of NHL patients treated with bendamustine-containing regimens, and potential risk factors were outlined, such as age ≥60 years old, number of bendamustine cycles, and nadir anti-CMV IgG levels. In addition, variations in the immune status were also described at baseline and during treatment.

CMV reactivation in NHL patients has been anecdotically reported after bendamustine exposure often in older subjects with iNHL including FL, MCL, or MALT [25,26,27,28,29,30,31,32]. Most of those cases are refractory or relapsed iNHL, and they usually received bendamustine with or without rituximab as second or above line of therapy developing CMV disease after the third cycle of treatment. Two case series of 30 and 38 older relapse or refractory iNHL have reported an incidence of 20 and 6% of CMV reactivation after bendamustine exposure, respectively [26,27]. Some risk factors have also been identified, such as CD4/CD8 ratio and anti-CMV IgG levels; however, the small number of patients who experienced CMV reactivation in each cohort, the great heterogeneity in type of disease, stage, and previous treatments, and lack of data on younger adults and/or subjects diagnosed with aggressive NHL treated with bendamustine as first-line therapy could not allow the identification of univocal predictors of CMV reactivation. In this study, we presented results on a large cohort of patients with both iNHL and aggressive diseases mostly treated with bendamustine-containing regimens as first-line therapy. No differences in incidence of CMV reactivation were registered between patients with iNHL or aggressive lymphomas at any stage, suggesting that viral reactivation might be more likely related to immune impairments caused by chemotherapeutic agents than to those induced by the disease itself. Indeed, patients who received RDB as first-line therapy experienced CMV reactivation more frequently than those receiving only bendamustine or RB or than patients who received bendamustine-based regimens as second or above line of treatment [26]. These differences might be linked to a more profound immunosuppression caused by synergistic effects on both adaptive and innate immune responses of the combination of dexamethasone, rituximab, and bendamustine; while other bendamustine-containing regimens, such as RB, might not markedly impair immune system. Several studies have already described that combination of rituximab and dexamethasone has additive effects by reducing tumor cell growth, increasing apoptosis and rituximab-mediated complement-dependent cytotoxicity, and influencing or delaying immune reconstitution [33,34,35,36,37]. Bendamustine might enhance these synergistic effects and increase immunosuppression [33]. Indeed, incidence of CMV reactivation was also increased in patients on RB protocol and was similar between RB as first line of therapy and RB administered as second or above line of treatment, as already reported in several case reports of refractory or relapse iNHL receiving RB as second or above line therapy.

Older age (>65 years) has been indicated as an important risk factor of CMV reactivation in kidney (HR = 2.43) and allogeneic hematopoietic stem cell transplant (HSCT; aged >50 years old; HR 1.40; 95% CI, 1.24–1.58) recipients [38,39,40]. In HSCT patients, CMV reactivation increases with T cell depletion and long-course steroid treatment [39]. In addition, in haploidentical HSCT, T cell repletion and post-transplant cyclophosphamide also augment the risk of CMV reactivation [41]. In NHL, CMV reactivation is a frequent cause of mortality, and prophylaxis might be warranted in patients at risk, such as patients with high antigenemia burden, recurrent CMV reactivation, and antiviral-associated toxicities [24]. In older iNHL (FL, MZL, and Waldenström macroglobulinemia) treated with bendamustine-containing regimens, the risk of CMV reactivation is increased (HR, 3.98; 95% CI, 1.40–11.26) compared to patients treated without bendamustine [18]; in particular, CMV reactivation is frequent in patients treated with bendamustine as third line or above therapy and exposed to corticosteroids [25,26,27,28,29,30,31,32]. Similarly, in our cohort, incidence of CMV reactivation was higher in patients aged >60 years; however, similar rates of reactivation were documented between indolent and aggressive diseases, or between bendamustine course as first-line therapy and second-line or above treatment (26 vs 11% respectively; *P* = 0.1361). Thus, we showed that CMV reactivation might complicate clinical courses and outcomes of both newly diagnosed and relapse/refractory older NHL patients with aggressive or indolent diseases.

CMV reactivation develops early in bendamustine administration usually after the third cycle of therapy, as documented in several case reports [25,26,27,28,29,30,31,32]; however, no significant differences in long-term cumulative incidence of reactivation were registered in our cohort between patients who received less than three courses of bendamustine and subjects who had more than three cycles of therapy, while in multivariate analysis the number of cycles of bendamustine increased the risk of CMV reactivation in treatment-naïve patients who received bendamustine as first-line therapy. This augmented susceptibility to viral reactivation might be caused by an impairment in T cell immunity, such as decreased circulating levels of CMV-specific CD8^+^ cytotoxic lymphocytes and CD4^+^ T cells [19,28,31,34,42,43]. In our study, patient with CMV reactivation had a significant depression of CD4^+^ T cell count between the second and third cycle of bendamustine compared to those subjects without CMV reactivation, in contrast with Isono et al. who documented a CD4^+^ cell decreased after the sixth course of bendamustine [26,43]. CD4^+^ T cells are important in maintaining CMV in its latent form, as a lack of CMV-specific CD4^+^ T cells is associated with a persistent viral shedding into urine and saliva even in healthy young subjects [44]. In addition, CMV-specific CD8^+^ T cells require the support of CMV-specific CD4^+^ T cells to prevent CMV reactivation after HSCT [45]. Therefore, CD4^+^ T cell depression during bendamustine treatment might affect the CMV-specific CD4^+^ T cell pool, thus increasing susceptibility to CMV reactivation in NHL patients. We also documented a significant decrease in circulating anti-CMV IgG at the nadir of reactivation which likely mirrors hyperactivation and/or exhaustion in both B and T cell immune responses [28].

Clinical manifestations and severity of CMV reactivation vary among patients treated with bendamustine, from no symptoms to severe CMV disease and death [25,26,27,28,29,30,31,32], as also described in our cohort. In symptomatic patients, various antiviral agents are employed, such as ganciclovir and VGCV at 900 or 1,800 mg/daily [46]. VGCV has also shown efficacy in prophylaxis of CMV reactivation in allogeneic HSCT at low dose (450 mg/daily for six months); however, drug-related toxicity, especially myelosuppression, might require drug discontinuation [47]. In our cohort, 18 patients who experienced CMV reactivation and high viremia with or without symptoms were treated with VGCV at low or high dose showing a median time of CMV viremia negativization of 45.5 days (range, 17–152 days) and four CMV disease-related deaths. No drug-related adverse events requiring antiviral agent discontinuation were registered.

In conclusion, CMV reactivation is a threat in hematologic patients who undergo chemotherapy for hematologic malignancies and/or conditioning regimens for HSCT [24,40]. Bendamustine, an alkylating agent, is used for treatment of iNHL; however, secondary infections are anecdotically reported, such as CMV reactivation in relapse/refractory iNHL patients [25,26,27,28,29,30,31,32]. In our study, we described an increased incidence of CMV reactivation in older adults (age >60 years old) with newly diagnosed and relapse/refractory iNHL and aggressive disease treated with bendamustine-containing regimens, especially after the third course of bendamustine accompanied by a significant depression of circulating CD4^+^ T cell count and anti-CMV IgG levels. Therefore, a close and early monitoring of CD4^+^ T cell frequency, CMV-DNA, and virus-specific IgG levels might be required in older hematologic patients who received bendamustine, especially in combination with rituximab and dexamethasone, to prevent CMV reactivation and improve clinical management and outcomes of those subjects.
